# Mesoporous Potassium-Based Metal–Organic Framework as a Drug Carrier

**DOI:** 10.1021/acscentsci.5c00904

**Published:** 2025-07-25

**Authors:** Bo Fang, Tianyu Shan, Sailing Chen, Fei Pan, Xue Yang, Ding Xiao, Feihe Huang, Zhengwei Mao

**Affiliations:** † Department of Hepatobiliary and Pancreatic Surgery, The Second Affiliated Hospital, School of Medicine, 12377 Zhejiang University, Hangzhou 310003, China; ‡ Stoddart Institute of Molecular Science, Department of Chemistry, Zhejiang University, Hangzhou 310058, China; § Zhejiang-Israel Joint Laboratory of Self-Assembling Functional Materials, ZJU-Hangzhou Global Scientific and Technological Innovation Center, Zhejiang University, Hangzhou 311215, China; ∥ Department of Reproductive Endocrinology, Women’s Hospital, School of Medicine, Zhejiang University, Hangzhou 310006, China; ⊥ State Key Laboratory of Resource Insects, Institute of Apicultural Research, Chinese Academy of Agricultural Sciences, Beijing 100093, China; # MOE Key Laboratory of Macromolecular Synthesis and Functionalization, Department of Polymer Science and Engineering, Zhejiang University, Hangzhou 310058, China

## Abstract

To date, the number of reported mesoporous metal–organic frameworks (MOFs) remains limited. Herein, we report a novel mesoporous potassium-based MOF (K-MOF), designated as **KMOF-1**, whose precise structure was determined by using single-crystal X-ray diffraction. **KMOF-1** used 18-crown-6 units as the organic linkers and potassium ions as the metal centers, forming a framework topological structure with interconnected four-membered rings. The specific surface area of the synthesized **KMOF-1** was determined by the Brunauer–Emmett–Teller method, which showed a high specific surface area of 1034 m^2^/g. **KMOF-1** was demonstrated to be a promising drug carrier, exhibiting encapsulation capabilities for various drugs and maintaining stability for a defined period under simulated physiological conditions. Using vascular endothelial growth factor (VEGF) aptamers as model drugs, we further confirmed the effective loading of VEGF aptamers in **KMOF-1** (**KMOF-1@VEGF**) and the ability of **KMOF-1@VEGF** to release VEGF aptamers responsively in acidic environments. Additionally, in vitro studies showed that **KMOF-1** protected VEGF aptamers from degradation by nucleases, allowing them to be effectively taken up by cells. This novel K-MOF, with its biocompatible metal centers, mesoporous channels, and demonstrated efficacy as a drug carrier, offers a significant advancement in developing MOF-based drug delivery systems.

## Graphical abstract

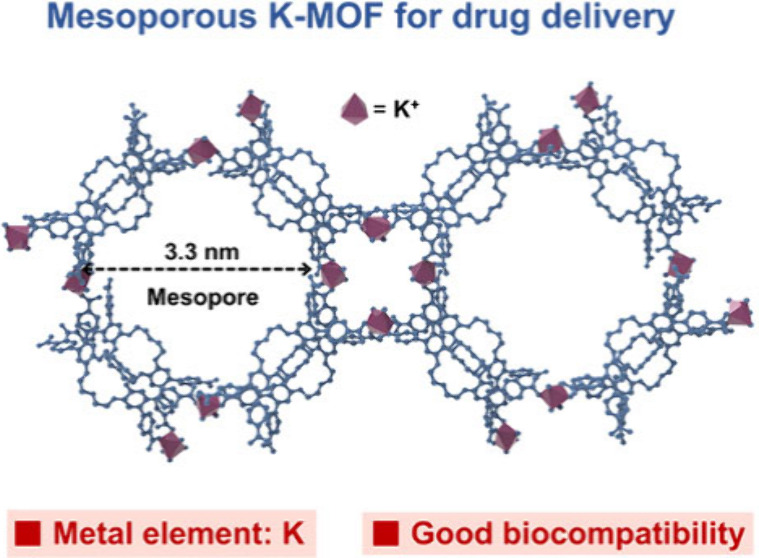

## Introduction

Metal–organic frameworks (MOFs) are a category of porous materials created through the coordination of metal ions or metal clusters with organic ligands via self-assembly.^1,2^ This combination of metal ions and organic ligands results in a novel organic–inorganic hybrid framework that possesses a periodic network structure with well-defined spatial geometry.^3^ Compared to traditional porous materials, MOFs present several advantages, such as high porosity, large specific surface area, tunable pore size, structural diversity, and the potential for multifunctional modification.^4^ Furthermore, current research reveals that some MOFs display enzyme-like activity.^5^ Due to these advantages, MOF-based materials hold significant promise for applications in drug loading and delivery. In general, MOFs can be mainly categorized based on pore size into microporous MOFs (with pore sizes smaller than 2 nm) and mesoporous MOFs (with pore sizes ranging from 2 to 50 nm). Microporous MOFs, with their limited pore size, are predominantly utilized for accommodating small molecules, whereas mesoporous MOFs, with the ability to incorporate larger biomacromolecules, are regarded as excellent carriers for drug delivery and have attracted widespread research attention in recent years.^6^ However, among the more than 100,000 reported MOFs, only a minuscule fraction of MOFs (<0.03%) possess mesoporous structures.^7,8^ The formation of a mesoporous structure is thermodynamically unfavorable compared to their denser counterparts, rendering the synthesis of mesoporous MOFs a considerable challenge.

In addition to limitations related to pore size, the potential toxicity of current drug delivery systems based on MOFs presents a significant obstacle to their clinical applications. Previous research indicates that the primary metal elements within MOFs influence their overall toxicity.^9^ To design safe MOFs for biomedical applications, the type of metals should be carefully considered.^10^ Currently, most MOFs used for drug delivery use transition metals as nodes. Although several strategies have been developed to reduce the toxicity, such as surface modification, these methods remain insufficient in preventing the toxicity associated with the long-term accumulation of heavy metals in the body. K, as the second most abundant metal element in living organisms, exhibits good tolerance in the body. Utilizing the K element to design and synthesize MOFs for drug delivery is anticipated to overcome the inherent metal toxicity of traditional MOFs. Cyclodextrin-based MOFs with K^+^ as the metal nodes were reported with excellent drug loading capacity and high biocompatibility.^11^ In addition, the potassium-based MOF (K-MOF) synthesized from the pery­lene-3,4,9,10-tetra­carb­oxy­late linker also showed interesting applications and can be used as a humidity sensor to achieve precise humidity threshold monitoring.^12^ However, the currently reported K-MOFs are still limited and have restricted pore sizes.

Crown ethers are macrocyclic compounds characterized by ether oxygen atoms as repeating units.^13^ The ionization properties of crown ethers render them well-suited for transmembrane transports and interaction with various biological systems.^14^ Numerous research teams have investigated the roles of crown ethers as drug delivery vehicles,^15^ ion transport carriers,^16^ ion channels,^17^ drug-targeting carriers,^18^ and nanocarriers.^19^ Furthermore, existing studies indicated that the presence of crown ethers during drug delivery enhanced drug permeability and solubility while reducing toxicity.^20^ The cavity of crown ethers can reversibly bind with diverse metal ions. Some studies indicate that controlling the pH and solvent conditions can promote the coordination of metal ions with carboxyl-containing crown ethers. Lower metal ion concentrations are more likely to yield ordered structures.^21–23^ However, the intrinsic flexibility of crown ether molecules, coupled with the high and variable coordination numbers of the potassium metal nodes, has rendered the syntheses of crown ether-containing K-MOFs and the determination of their single-crystal structures highly challenging.

In this work, we report the synthesis of a novel K-MOF, termed **KMOF-1**, which has a well-defined mesoporous structure characterized by single-crystal X-ray diffraction (SCXRD). **KMOF-1** has 18-crown-6 organic linkers and K^+^ metal centers, exhibiting ordered, noninterpenetrated mesoporous channels (Figure 1). Besides, it showed a high specific surface area of 1034 m^2^/g. Given the ordered pore channels and high specific surface area of **KMOF-1**, we further explored its potential for drug loading. The vascular endothelial growth factor (VEGF) aptamers were identified as suitable model drugs through a drug loading screening process using **KMOF-1** with various drugs. The VEGF aptamers loaded in **KMOF-1** can be protected from nuclease degradation and effectively taken up by cells. This work demonstrates that **KMOF-1** is an excellent drug carrier, providing a further reference for the development and utilization of highly biocompatible mesoporous MOFs.

**Figure 1:**
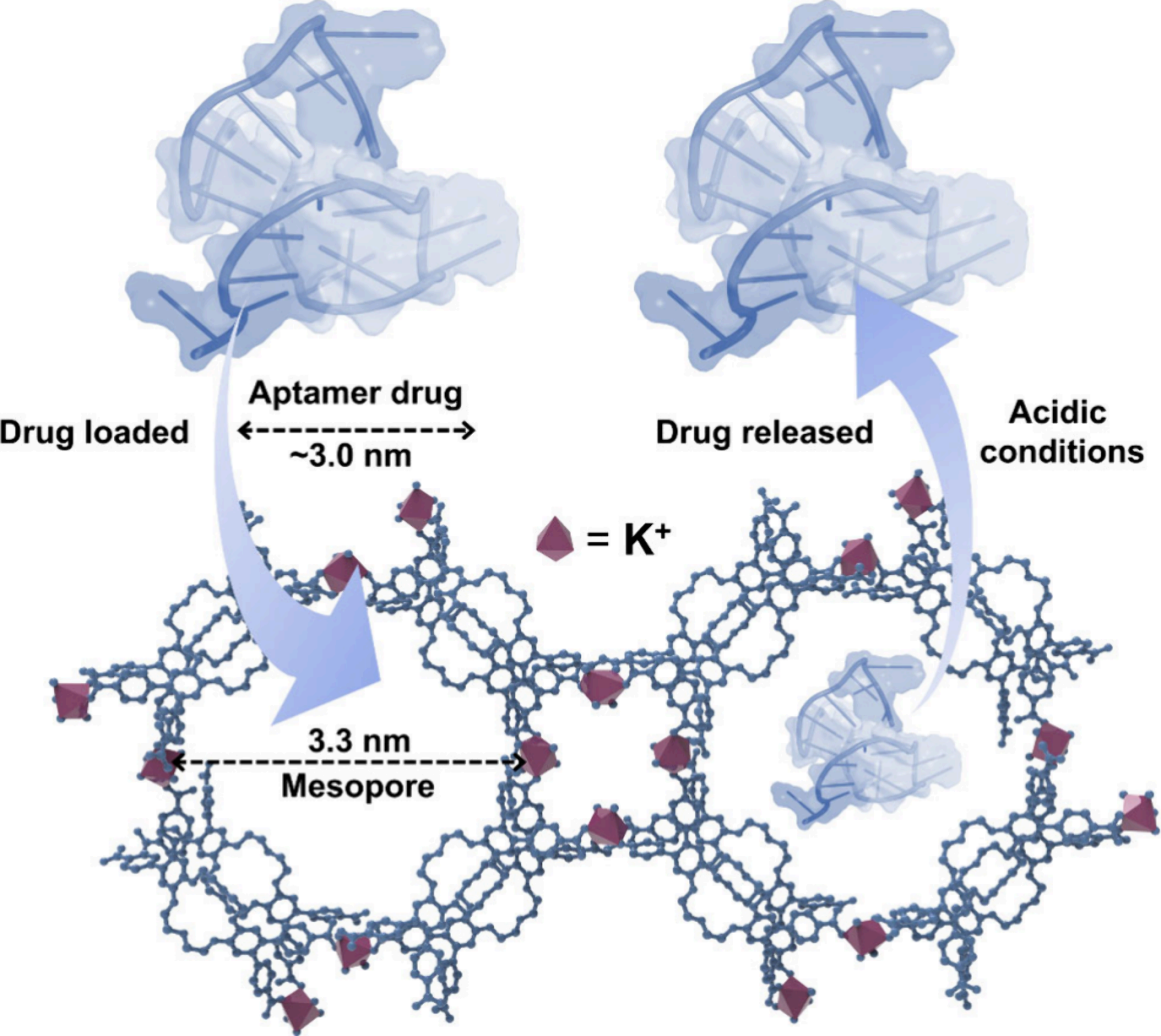
Mesoporous K-MOF synthesized in this study, featuring potassium ions as the metal centers and 4,4′5,5′-terabenzoic acid dibenzo-18-crown-6 units as the organic linkers, with unique dual-pore channels (1.2 nm micropores and 3.3 nm mesopores). With VEGF aptamer as a model drug, **KMOF-1** demonstrated excellent drug loading capability and drug release ability under acidic conditions.

## Results and Discussion

### Crystal Structure of KMOF-1

**KMOF-1** was prepared by synthesizing 4,4′5,5′-terabenzoic acid dibenzo-18-crown-6 units and recrystallizing them by slow evaporation. The recrystallization employed a mixed solution of *N*,*N*-dimethylformamide (DMF), H_2_O, and ethanol (v/v = 4/2/1) at 85 °C, yielding colorless crystalline **KMOF-1**. Single-crystal X-ray diffraction (SCXRD) analysis confirmed that **KMOF-1** crystallizes in the tetragonal space group *I*4̅, revealing its highly ordered structure (Table S1). In the framework, potassium ions served as the metal centers, while crown ether units acted as organic linkers, collectively constructing **KMOF-1**. This framework exhibited a topological structure characterized by four-membered rings and one-dimensional pore channels (Figure 2a). **KMOF-1** possessed two types of channels: one with a diameter greater than 3.0 nm and another approximately 1.2 nm in diameter. These channels alternated periodically without interpenetration, which may facilitate efficient drug adsorption and release (Figure 2b). At the connection nodes, the coordination environment of K^+^ with the carboxylate groups is illustrated in Figure 2c, where one K^+^ ion was coordinated by four carboxylate fragments and two water molecules. Notably, one carboxylate group existed in its deprotonated form, ensuring the overall charge neutrality of the framework without additional charged solvent molecules. Observations along one axis of the structure revealed layered stacking and an open channel system (Figure 2d,e).

**Figure 2:**
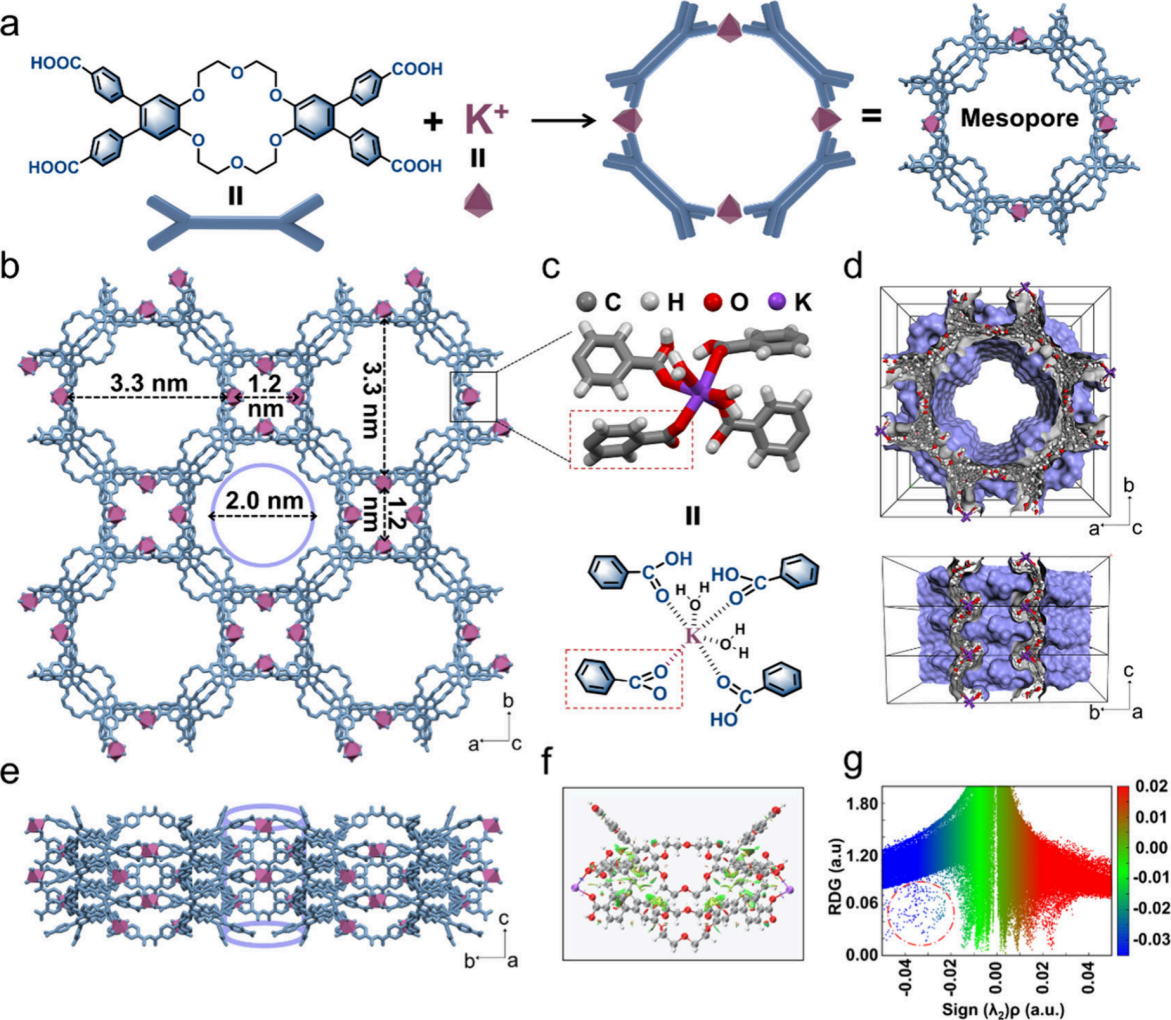
Synthesis and crystal structure of **KMOF-1**. (a) Schematic illustration of the formation of **KMOF-1** from potassium ions and crown ether units, resulting in the formation of four-membered rings that assemble into a mesoporous framework. Lattice water molecules are omitted for clarity. (b) Top view of the **KMOF-1** structure, highlighting the mesoporous channels (diameter of ∼3.3 nm) and microporous channels (diameter of ∼1.2 nm). (c) Coordination environment of K^+^ within **KMOF-1**, showing the coordination of K^+^ with water molecules and carboxylate groups from the crown ether units. (d and e) Views of the **KMOF-1** structure along different crystallographic axes, illustrating the open channel system and layered stacking arrangement. (f) RDG isosurface plot visualizing noncovalent interactions between stacked crown ether molecules within the **KMOF-1** framework. (g) RDG scatter plot illustrating the types and strengths of noncovalent interactions within the **KMOF-1** framework.

To investigate the noncovalent interactions presented in **KMOF-1**, we performed a reduced density gradient (RDG) analysis on the locally stacked upper and lower units of **KMOF-1**. As shown in Figure 2f, the RDG isosurface plot revealed various noncovalent interactions between the two stacked crown ether molecules. In particular, there were strong attractive interactions (blue regions) near the carbonyl oxygen of the carboxylate group coordinated with K^+^. The green regions indicated that van der Waals forces were widely distributed between the two stacked systems, while some steric effects can be observed near the benzene rings (red regions). The results of the RDG scatter plot (Figure 2g) further demonstrated the presence of strong attractive interactions between the two crown ether molecules (red circles). The electrostatic potential (ESP) analysis of **KMOF-1** revealed that the ESP between the upper and lower stacking layers within the framework was not homogeneously distributed around the crown ether molecules (Figure S1). Instead, it follows an intricate spatial pattern. This distinct ESP distribution was presumably strongly associated with the geometric structure and electron density distribution of the **KMOF-1**. We further conducted electron localization function (ELF) analysis on the upper and lower stacking layers of **KMOF-1** to elucidate the distribution patterns and interaction characteristics of electrons within **KMOF-1** (Figure S2). The relatively high electron density surrounding the potassium atoms, along with a degree of electron localization, was likely associated with the metallic character of potassium and its chemical state within the compound. Moreover, as shown in Figures S3–S6, we conducted a detailed analysis and visualization of the electronic structure and interaction characteristics of **KMOF-1**’s internal left and right stacking layers through ESP analysis, RDG analysis, and ELF analysis. Similar stacking patterns were observed in the local lateral layers of **KMOF-1** as compared to the upper and lower layers. The strong attractive forces surrounding K^+^ helped maintain the ordered structure and stability of **KMOF-1**. Currently, only a limited number of K-MOFs have been reported with micropores (Table S2). Therefore, we have synthesized a K-MOF with mesopores, which has potential application as a drug delivery carrier.

### VEGF Aptamers Loading into **KMOF-1** as Model Drugs

Previous studies have suggested that drug-loaded MOFs should be stable under physiological conditions to deliver target molecules to tissues while also being degradable and easily eliminated from the body without endogenous accumulation.^25^ To explore the potential of **KMOF-1** as a drug carrier, we further conducted morphological characterization and stability analysis on it. As shown in Figure 3a, scanning electron microscopy (SEM) characterization revealed that **KMOF-1** has a bulky morphology. Further elemental analysis confirmed the presence of C, O, and K on its surface (Figure 3b). We examined the X-ray diffraction (XRD) changes in **KMOF-1** after exposure to different solvents for a certain time. As shown in Figure S7, the results indicated that **KMOF-1** retained its crystalline structure after exposure to water, culture medium, and PBS buffer (pH 7.4 or 6.5), demonstrating a certain level of stability. The nitrogen adsorption isotherm of **KMOF-1** showed a type IV curve, indicating the presence of mesopores, and its specific surface area was as high as 1034 m^2^/g (Figure 3c). These findings suggest that **KMOF-1** is a potential drug carrier. To study the drug loading capacity of **KMOF-1**, we established UV absorption standard curves for various drugs (Figures S8–S19) and assessed its loading performance on different drugs by using the immersion method. The results showed that **KMOF-1** effectively loaded diverse drug molecules, including small-molecule drugs like quercetin and large-molecule drugs like VEGF aptamers (Figures S20 and S21). In the initial screening, **KMOF-1** exhibited the best loading efficiency for the VEGF aptamers. Notably, some larger molecules, such as insulin (INS) and hemoglobin (HGB), could also be loaded by **KMOF-1**. Previous studies have shown that some biopolymers with flexible structures and dynamic conformational changes can be effectively encapsulated in MOFs, even if their sizes are larger than the pore size of the MOFs.^26^ Besides, it is also reasonably postulated that the load of INS and HGB on **KMOF-1** might be attributed to the dissociation of INS and HGB (existing in polymeric forms) or the presence of their monomers in a partial manner (size of insulin monomer: ∼1.3 nm). Collectively, these findings further supported **KMOF-1** as a versatile drug carrier.

**Figure 3:**
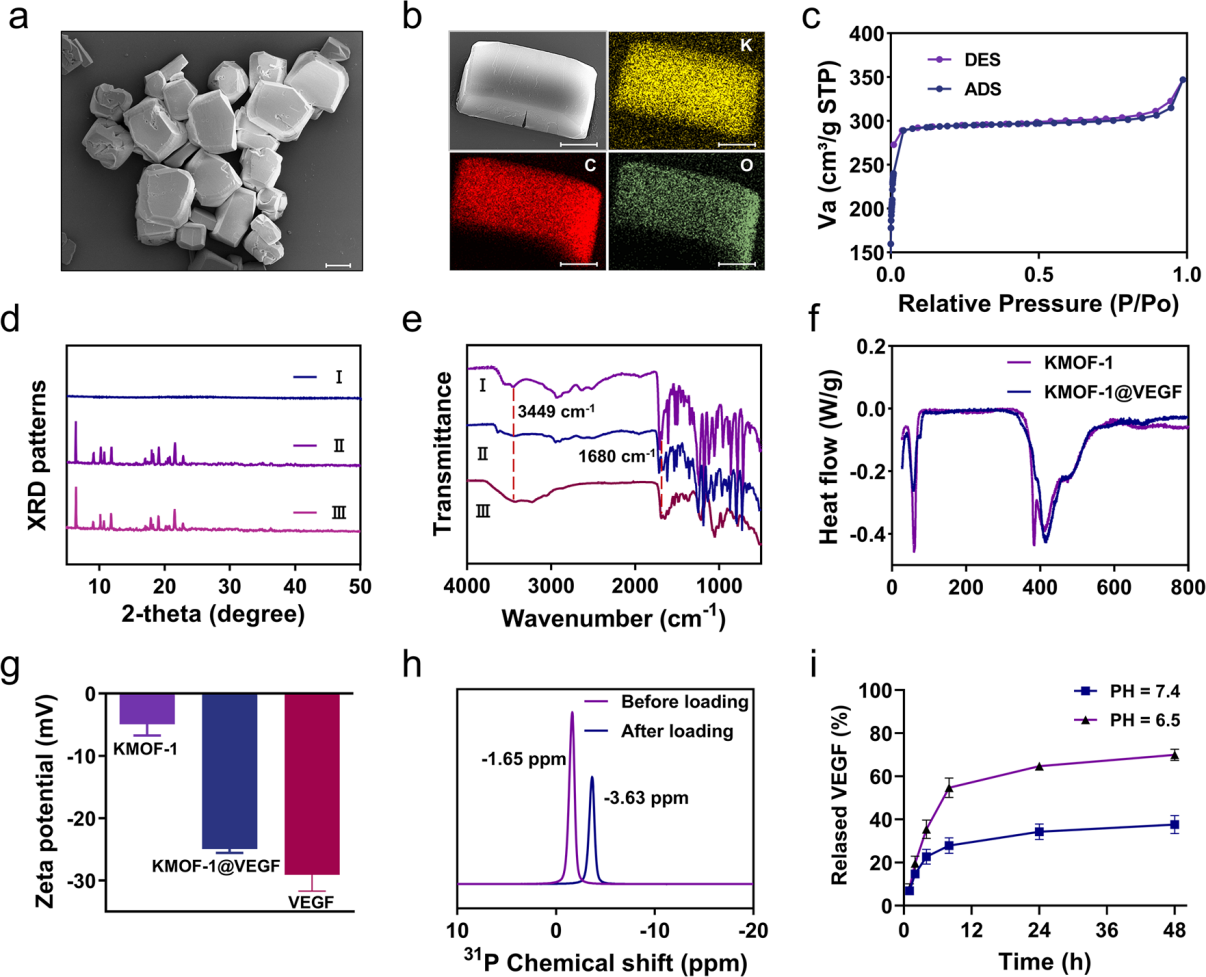
Application of **KMOF-1** in the loading of drugs. (a) SEM image of **KMOF-1**. Bars indicate 10 μm. (b) Elemental mapping analysis of **KMOF-1**, confirming the presence of C, O, and K elements. Bars indicate 5 μm. (c) Nitrogen adsorption and desorption curves of **KMOF-1**. ADS = adsorption and DES = desorption. (d) XRD patterns of VEGF, **KMOF-1**, and **KMOF-1@VEGF**, demonstrating the preservation of the crystal structure after drug loading. I: VEGF; II: **KMOF-1**; III: **KMOF-1@VEGF**. (e) FTIR spectra of VEGF, **KMOF-1**, and **KMOF-1@VEGF**. I: **KMOF-1@VEGF**; II: **KMOF-1**; III: VEGF. (f) DTG curves of **KMOF-1** and **KMOF-1@VEGF**, showing enhanced thermal stability of **KMOF-1** upon VEGF aptamer loading. (g) Zeta potential of VEGF, **KMOF-1**, and **KMOF-1@VEGF**, indicating a change in surface charge upon VEGF aptamer loading. (h) Solid-state ^31^P NMR spectra of VEGF (before loading) and **KMOF-1@VEGF** (after loading). (i) In vitro release profiles of VEGF aptamers from **KMOF-1@VEGF** at pH 7.4 and 6.5, showing a pH-responsive release behavior.

Given the superior loading efficiency observed for the VEGF aptamers, we selected them as the model drugs for subsequent investigations of the loading mechanisms of **KMOF-1** (**KMOF-1@VEGF**). As shown in Figure S22, SEM characterization further confirmed that **KMOF-1@VEGF** displayed irregular surfaces. Further elemental analysis indicated the presence of N and P elements, confirming the successful loading of VEGF onto **KMOF-1** (Figure S23). Moreover, XRD analysis indicated no significant changes in the crystal structures before and after drug loading, demonstrating the integrity of the internal structure of **KMOF-1@VEGF** (Figure 3d). Additionally, a comparison of infrared spectroscopy revealed absorption peaks at 3449 and 1680 cm^–1^ in **KMOF-1@VEGF**, corresponding to −NH vibration absorption peaks and base-related absorption peaks in the VEGF aptamers, respectively (Figure 3e). This result also indicated the successful loading of the VEGF aptamers. Thermogravimetric analysis (TGA) was conducted from ambient temperature up to 800 °C; as shown in Figure 3f, a rightward shift in thermal denaturation curves for **KMOF-1@VEGF** was observed, indicating improved thermal stability after drug loading compared to standalone **KMOF-1**. Furthermore, the zeta potential results indicated that the zeta potential of drug-loaded **KMOF-1@VEGF** was −24.9 mV, further supporting both drug loading and enhanced stability after loading (Figure 3g). To further confirm the successful loading of VEGF into **KMOF-1**, we conducted solid-state NMR spectroscopy on VEGF and **KMOF-1@VEGF**. As shown in Figure 3h and Figure S24, the phosphorus signal of VEGF appeared at −1.65 ppm. After loading into **KMOF-1**, the phosphorus signal of **KMOF-1@VEGF** shifted upfield compared to free VEGF, indicating enhanced shielding within the **KMOF-1** framework. This observation suggested strong interactions between VEGF and **KMOF-1**, supporting the effective encapsulation of VEGF within the framework.

By optimizing the mass ratios (1:2), we achieved a high drug loading capacity for **KMOF-1@VEGF**, with the maximum loading capacity reaching 52.9% (Figure S25). We also examined the drug release behavior of **KMOF-1@VEGF** under different pH conditions. As shown in Figure 3i, **KMOF-1@VEGF** exhibited higher release efficiency under weakly acidic conditions, with a drug release rate approaching 70.3% within 48 h. Reported drug-loaded MOFs achieve pH-responsive drug release mainly via several mechanisms: (1) MOF structural disintegration; (2) ligand protonation/deprotonation; and (3) ion exchange.^27–30^ **KMOF-1** was stable in weakly acidic conditions (Figure S7, V) but also degraded into smaller particles under strong acid conditions (pH < 2) as shown in Figure S26. These results indicated that **KMOF-1** may release drugs via different mechanisms in different acidic environments. Therefore, **KMOF-1** can serve as a good drug carrier capable of efficiently loading aptamer drugs and achieving pH-responsive release.

### Drug Loading Simulation

We further conducted molecular dynamics (MD) simulations to understand the kinetic process of the VEGF aptamers loading onto **KMOF-1**. The results of the MD simulations demonstrated that the stepwise adsorption mechanism of **KMOF-1** for VEGF aptamers was consistent with the diffusion mechanism inferred from fitting the diffusion patterns (Figure 4a). As previously noted, **KMOF-1** contained two types of channels: one was a mesoporous channel with a diameter of 3.3 nm, and the other was a microporous channel with a diameter of 1.2 nm. After constructing a 2 × 2 × 3 supercell for **KMOF-1**, we performed grand canonical Monte Carlo (GCMC) calculations to explore the adsorption pathway of **KMOF-1** by gradually increasing the number of VEGF aptamer molecules. Figure 4b showed that due to volume limitations and steric hindrance, VEGF aptamer molecules mainly occupied the mesoporous channels of **KMOF-1**. Further analysis of the interaction between a single VEGF aptamer molecule and **KMOF-1** (as shown in the black dashed box) indicated that the VEGF aptamers interacted with the framework of **KMOF-1** through hydrogen bonds (red circles), forming a tightly adsorbed structure.

**Figure 4:**
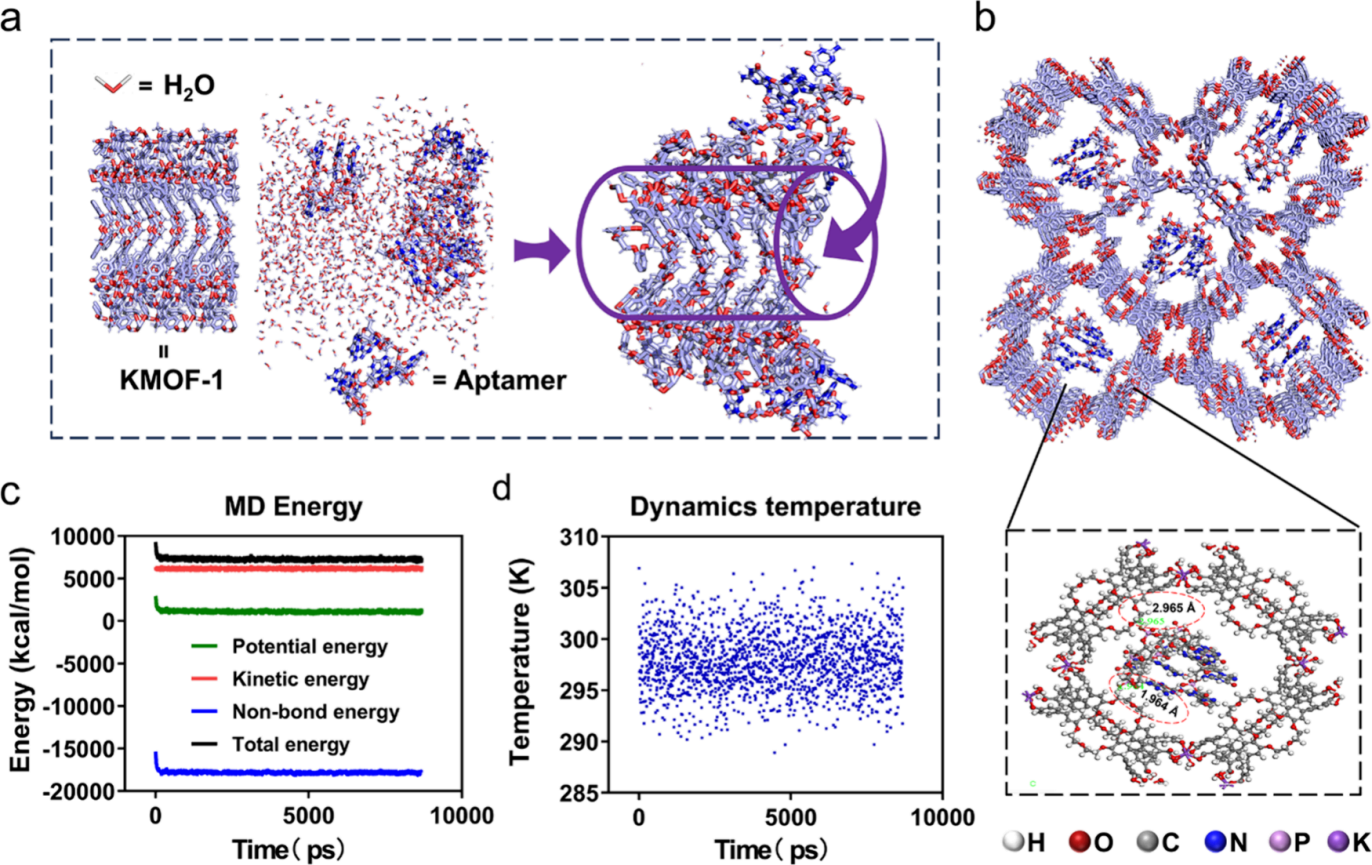
MD simulations of VEGF aptamers loading into **KMOF-1**. (a) Schematic representation of the stepwise adsorption process of VEGF aptamer molecules into the mesoporous channels of **KMOF-1**. (b) The zoomed-in region depicts the location and interaction of the VEGF aptamer molecules within the **KMOF-1** framework. (c) Energy profiles obtained during the MD simulation, showing the potential energy, kinetic energy, nonbonded energy, and total energy of the system as a function of simulation time. (d) Temperature fluctuations during the MD simulation, indicating a stable thermal environment throughout the simulation.

In addition, as shown in Figure 4c, at the beginning of the simulation, the kinetic energy (red curve) fluctuated around approximately 6000 kcal/mol, which indicated that throughout the simulation stage, the movement speeds of the particles tended to be stable. The potential energy (green curve), on the other hand, showed slight fluctuations in the initial stage and then stabilized at a level close to 0 kcal/mol. This suggested that there was no substantial change in the potential energy after the interactions between the molecules. Notably, both the total energy (black curve) and the nonbonded energy (blue curve) decreased sharply in the initial stage and then gradually tended to stabilize. This indicated that the system had undergone significant energy adjustments in the initial stage, especially with the involvement of noncovalent interactions, before reaching a relatively stable energy state. The changes in these energy curves were in line with the general laws of MD simulations. In the initial stage of the simulation, the system needed to be adjusted to achieve equilibrium, so obvious energy changes would occur before stabilization. Subsequently, the total energy rapidly decreased before stabilizing with minor fluctuations. The nonbonded energy quickly adjusted and then stabilized, while the potential and kinetic energies fluctuated within certain ranges. From these changes, it can be observed that stable binding between **KMOF-1** and VEGF aptamers was primarily formed through van der Waals forces and electrostatic interactions without significant chemical bond breakage or formation within the system. In Figure 4d, the blue points represent temperature fluctuations during the MD process. The temperature fluctuated between 290 and 305 K without any significant upward or downward trend, indicating that throughout the simulation process, the temperature remained relatively stable without substantial variations. This suggested that the thermal environment of the simulated system was stable. Therefore, our MD simulation results indicated that **KMOF-1** can effectively load VEGF aptamers through noncovalent interactions.

### Protection and Uptake of VEGF Aptamers

To explore the drug delivery effect of **KMOF-1**, we first evaluated the protective effect of **KMOF-1** on VEGF aptamers by immersing **KMOF-1@VEGF** in 10% fetal bovine serum (FBS) solution to simulate the nuclease environment (Figure 5a). In the absence of **KMOF-1**, the band intensity in the DNA gel electrophoresis experiment of the VEGF combined FBS group decreased significantly, indicating that the VEGF aptamers were almost completely degraded due to the action of nucleases in the FBS solution. However, after immersing **KMOF-1@VEGF** in FBS solution for 1 h (**KMOF-1@VEGF** + FBS group), the band of VEGF aptamers still existed, which showed that **KMOF-1** had an excellent antidegradation protective effect on the VEGF aptamers. By using Cy3 dye to fluorescently label the VEGF aptamers (Cy3-VEGF), we further analyzed whether the VEGF could be released from **KMOF-1** and effectively taken up by cells. As shown in Figure 5d, after incubating the Cy3-labeled **KMOF-1@VEGF** with the colon cancer cells HCT116 for 2 h, Cy3-VEGF aptamers were observed to be distributed in the cell membrane, nucleus, and cytoplasm, indicating that the VEGF aptamers encapsulated in **KMOF-1** can be effectively taken up by cells. Notably, we observed Cy3-VEGF aptamers also in the nucleus, likely due to the passive diffusion of smaller aptamers through the lipid bilayer of the nuclear membrane.^31^ Besides, to assess the biocompatibility of **KMOF-1@VEGF**, we carried out a hemolysis assay. Our results showed that **KMOF-1@VEGF** did not show significant hemolytic effects (Figure 5b). Moreover, we also examined the toxicity of **KMOF-1@VEGF** in normal cells. As shown in Figure 5c and Figure S27, our results indicated that **KMOF-1@VEGF** exhibited low toxicity to normal cells, further demonstrating its good safety. The above results showed that **KMOF-1** was a safe and effective carrier, which can protect the encapsulated aptamers from degradation and enable their effective release.

**Figure 5:**
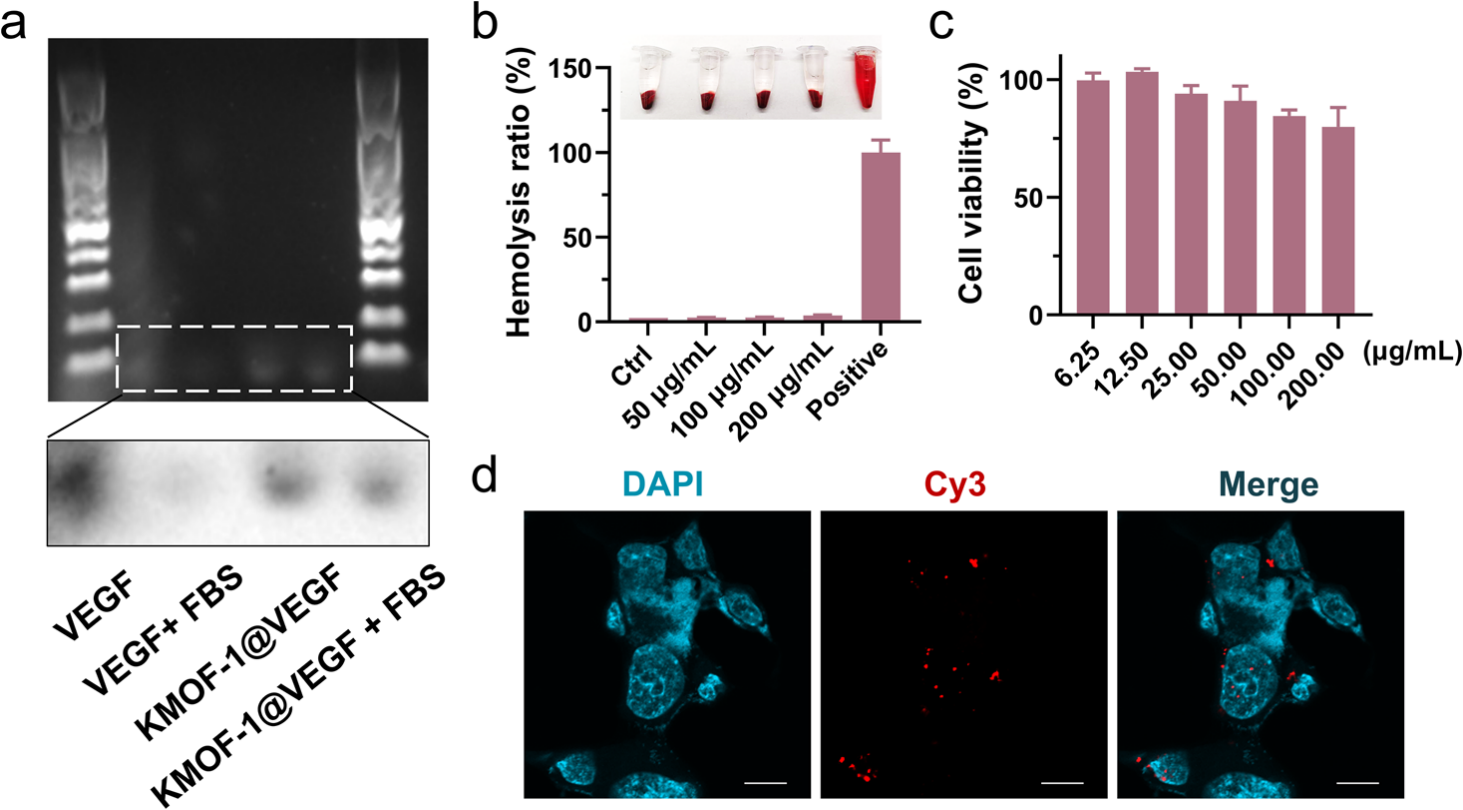
Protective and targeted release of VEGF aptamers by **KMOF-1**. (a) DNA gel electrophoresis analysis of VEGF aptamer degradation in 10% FBS solution with or without **KMOF-1** encapsulation. (b) Hemolysis rate of red blood cells treated with different concentrations of **KMOF-1@VEGF**. Positive: water. (c) Cytotoxicity of **KMOF-1@VEGF** on normal cell line AML12. Bars represent the mean ± SD of three independent experiments. (d) Confocal microscopy images showing the uptake of Cy3-labeled **KMOF-1@VEGF** by HCT116 cancer cells after 2 h of incubation. Blue: DAPI (nucleus). Red: Cy3-VEGF. Bars indicate 10 μm.

## Conclusion

In this report, we synthesized a mesoporous K-MOF, designated as **KMOF-1**, featuring a structure that exhibited an ordered open channel system. Computational studies confirmed that noncovalent interactions played a crucial role in stabilizing the **KMOF-1** framework. Drug screening with molecules of different sizes confirmed **KMOF-1** as a versatile drug carrier capable of encapsulating a wide range of therapeutic drugs. VEGF aptamers were selected as model drugs to further confirm the drug loading behavior and kinetics of **KMOF-1**. MD simulations demonstrated robust attractive interactions between the VEGF aptamers and the pore environment of **KMOF-1**, which dictated the gradual loading of the VEGF aptamers within **KMOF-1**. Furthermore, **KMOF-1** protected the VEGF aptamers from nuclease degradation, demonstrating favorable biocompatibility and facilitating efficient aptamer delivery. Our study expands the repertoire of mesoporous MOF materials, offering insights into the development and utilization of MOFs as drug delivery platforms.

## Supplementary Material



## Notes

Additional experimental details, materials, and methods; ESP, ELF and RDG analyses; XRD patterns of **KMOF-1** in different solvents; standard curves of different drugs; screening of model drugs for loading; SEM and elemental mapping images of **KMOF-1@VEGF**; NMR spectra; optimization of drug loading capacity of **KMOF-1**; cell cytotoxicity of **KMOF-1@VEGF**; crystal data; and comparison of different K-MOFs (PDF)
