# Supplementary material for: Mesoporous Potassium-Based Metal–Organic Framework as a Drug Carrier
Source: ACS Cent Sci. 2025 Jul 25;11(9):1651–8. doi: 10.1021/acscentsci.5c00904 (PMC12464762; doi:10.1021/acscentsci.5c00904)
Supplement: Supplementary file 1 [file oc5c00904_si_001.pdf]

Supporting Information

Mesoporous Potassium-Based Metal-Organic Framework as a Drug Carrier

Bo Fang†[a,b,c], Tianyu Shan†[b,c], Sailing Chen†[d], Fei Pan[e], Xue Yang[b,c], Ding Xiao[b,c], Feihe Huang*[b,c], Zhengwei Mao*[a,f]

[a] Dr. B. Fang, Prof. Z. Mao
Department of Hepatobiliary and Pancreatic Surgery, The Second Affiliated Hospital, School of Medicine, Zhejiang University, Hangzhou 310003, China
E-mail: zwmao@zju.edu.cn

[b] Dr. B. Fang, Dr. T. Shan, Dr. X. Yang, Dr. D. Xiao, Prof. F. Huang
Stoddart Institute of Molecular Science, Department of Chemistry, Zhejiang University, Hangzhou 310058, China
E-mail: fhuang@zju.edu.cn

[c] Dr. B. Fang, Dr. T. Shan, Dr. X. Yang, Dr. D. Xiao, Prof. F. Huang
Zhejiang-Israel Joint Laboratory of Self-Assembling Functional Materials, ZJU-Hangzhou Global Scientific and Technological Innovation Center, Zhejiang University, Hangzhou 311215, China

[d] Dr. S. Chen
Department of Reproductive Endocrinology, Women's Hospital, School of Medicine, Zhejiang University, Hangzhou 310006, China

[e] Dr. F. Pan
State Key Laboratory of Resource Insects, Institute of Apicultural Research, Chinese Academy of Agricultural Sciences, Beijing 100093, China

[f] Prof. Z. Mao
Laboratory of Macromolecular Synthesis and Functionalization, Department of Polymer Science and Engineering, Zhejiang University, Hangzhou 310027, China

[+] These authors contribute equally to this work.

*Corresponding authors. E-mails: fhuang@zju.edu.cn and zwmao@zju.edu.cn

Table of Contents

1. Materials and methods....................S3

2. Local ESP, RDG, and ELF analyses of **KMOF-1**....................S8

3. Stability of **KMOF-1** in different solvents....................S10

4. Standard curves of different drugs....................S11

5. Screening of model drugs for loadings....................S15

6. Morphological characterization and ssNMR of **KMOF-1@VEGF**....................S16

7. Optimization of the drug loading capacity of **KMOF-1**....................S17

8. Cell cytotoxicity of **KMOF-1@VEGF**....................S17

9. Details of crystal data....................S18

10. Data on drug encapsulation in KMOFs....................S19

11. References....................S19

1. Materials and methods

Reagents

The solvents and drug reagents were purchased from the Sigma-Aldrich Chemical Company (St. Louis, USA). The VEGF aptamers with or without a Cy3 label were designed and purchased from Sangon Biotech (Shanghai, China).

Characterization

The solid-state ^{31}P nuclear magnetic resonance spectroscopy was performed on Bruker Avance Neo 400WB (Bruker Corporation, MA, USA). The zeta potential was determined using a NanoZetasizer (Zetasizer Nano, Malvern, UK). The element mapping analysis and scanning electron microscopy (SEM) observations were performed using Scios2 Hivac (Thermo Fisher Scientific, MA, USA). Ultraviolet spectra (UV) and drug loading efficiency were measured using a UV−vis spectrophotometer (Hitachi, Tokyo, Japan). Fourier Transform Infrared (FTIR) spectra were measured using an infrared spectrometer (Thermo Fisher Scientific, MA, USA). Powder X-ray diffraction (PXRD) analysis was performed on SmartLab SE (Rigaku, Tokyo, Japan). Thermogravimetric analysis (TGA) was conducted with a thermogravimetric analyzer (Waltham, MA, USA). Laser confocal microscopy (Nikon Corporation, Tokyo, Japan) was used for fluorescent analysis. Single crystal X-ray data were collected on a Bruker D8 VENTURE at 193 K (λ = 1.34139 Å). The structures were solved by intrinsic phasing methods (SHELXT) and refined by full-matrix least-squares on F^2 using SHELXL[S1] in the OLEX2[S2] program package. All non-hydrogen atoms were refined with anisotropic thermal parameters and the hydrogen atoms were fixed at calculated positions and refined by a riding mode. SQUEEZE routine implemented on PLATON[S3] was used to remove electron densities corresponding to disordered solvent molecules in the crystal data.

Syntheses of KMOF-1 and KMOF-1@VEGF

4,4′5,5′-Terabenzoic acid dibenzo-18-crown-6 (≥98% purity) was purchased from Zhengzhou Alpha Chemical Co. Ltd. (Zhengzhou, China). The synthesis of **KMOF-1** was carried out via the solvent evaporation method. Specifically, 4,4′5,5′-terabenzoic acid dibenzo-18-crown-6

(25.0 mg, 29.7 μM) was dissolved in 12 mL of *N*,*N*-dimethylformamide (DMF)/ethanol/water (v/v, 4/1/2) solution. Potassium chloride (2.3 mg, 30.2 μM) and 100 μL dilute hydrochloric acid (0.1 M) were added to the aforementioned solution. Subsequently, the solution was allowed to undergo slow evaporation at a controlled temperature of 85 ℃ for 24 h and yield **KMOF-1** as colorless crystals. After cooling to room temperature, the crystals were isolated by filtration, then washed with DMF, water and dried at 40 °C for 12 h.To synthesize **KMOF1-@VEGF**, we used the impregnation method. The impregnation process involved suspending a VEGF aptamer (150.0 nM) and **KMOF-1** (100.0 μg/mL) in a 5 mL water/ethanol mixture. The mixture was stirred in the dark at room temperature at 1000 rpm for 4 h. The drug-loaded composite was separated by centrifuging at 7000 rpm for 10 min. Unloaded VEGF was removed by washing twice with deionized water. The final product consisted of a pink solid (Cy3-labeled **KMOF-1@VEGF**) and a white solid (**KMOF-1@VEGF**), which were dried overnight under vacuum at 40 °C. The concentration of VEGF aptamers and drug loading capacity were determined by UV absorption at 260 nm. The synthesis of **KMOF-1** loaded with different drugs was similar to that of **KMOF-1@VEGF**. Drug loading capacity was evaluated as referred to in a previous study.[S4]

VEGF release study

The release of VEGF aptamers from the composite material was evaluated at pH 7.4 and pH 6.5. Samples were incubated in phosphate-buffered saline (PBS) solutions at pH 7.4 for physiological conditions and pH 6.5 for acidic conditions, and aliquots were withdrawn at predetermined time intervals. The concentration of released VEGF aptamers in each aliquot was determined by measuring the UV absorbance at a 260 nm wavelength. The percentage of released VEGF aptamers was calculated relative to the total amount of VEGF aptamers initially loaded in the composite.

Stability test of KMOF-1

To evaluate the stability of **KMOF-1** under different solvent conditions, **KMOF-1** was immersed in water, PBS buffer solution (pH = 7.4 or 6.5), and culture medium for 24 h.

Subsequently, the remaining crystals were collected via centrifugation, washed with the mother liquor used for single-crystal cultivation to remove residual solvents, and then dried. The dried samples were subsequently subjected to XRD analysis.

N_2 adsorptions analysis

A Quantachrome Autosorb-IQ2 automatic volumetric device was used to collect N_2 adsorption isotherms at 77 K via a liquid nitrogen bath. For BET surface area analysis, x/v(1 - x) was plotted versus x, where x = P/P_0 (P_0 = 1 bar) and v is the volume of nitrogen adsorbed per gram of MOF at standard temperature and pressure. A strong correlation and a positive C constant were observed. The linear segment delineated by dashed lines provided the slope ($[c-1]/v_{mc}$) and y-intercept ($1/v_{mc}$), yielding the monolayer capacity, v_m. The surface area was calculated using $A = v_m \sigma_0 N_{AV}$, where σ_0 signifies the cross-sectional area of the adsorbate at liquid density (16.2 Å^2 for nitrogen) and N_{AV} denotes Avogadro's number.

Molecular dynamics (MD) simulation

To explore the drug adsorption mechanism in metal-organic frameworks (MOFs), we utilized both MD and Monte Carlo simulations in Materials Studio. With the *Forcite* module, we carried out geometric optimizations, keeping each model at a neutral charge. The energy convergence standard was set to 2.0×10^{-5} kcal/mol, and the force tolerance was 1.0×10^{-3} kcal/mol/Å. The Universal Force Field (UFF) was applied to model van der Waals interactions, while the Ewald summation method was used to depict electrostatic interactions. The MOF had a tetragonal crystal system and belongs to the $I\bar{4}$ space group. Lattice parameters were optimized by minimizing energy, with values of a = 44.9 Å, b = 44.9 Å, c = 9.87 Å, and $\alpha = \beta = \gamma = 90°$. The optimization procedure made sure that the framework was relaxed before adsorption and MD simulations. Adsorption simulations were conducted using the Sorption module within a 2 × 2 × 3 supercell of the framework. The UFF was used to calculate Van der Waals interactions between adsorbates and adsorbents. To guarantee the credibility of the simulations, 1.0×10^6 equilibration steps were carried out at 298 K. Subsequently, 1.0×10^7 production steps were carried out to generate a set of configurations representing both the adsorbates and the

adsorbents. For MD simulations, the *Forcite* module was employed to study the MOF within a 1 × 1 × 4 supercell, where the crystal was sliced along the (001) plane. A 120 Å vacuum layer was added to isolate the MOF from periodic images. Then, the system was filled with 1000 water molecules and 5 drug molecules using the Amorphous Cell module. The system went through equilibration *via* an 8.0 ns NVT (constant number of particles, constant volume, and constant temperature) simulation at 298 K and 1 atm. The UFF was used to model intermolecular interactions and atomic charges were obtained from the QEq method. Electrostatic and van der Waals interactions were calculated using the Ewald summation method.

Calculations

All the calculations were conducted using the Gaussian 16, C01 software package. For all the calculations, the PBE0 hybrid functional was employed in conjunction with the D3 version of Grimme's dispersion with Becke-Johnson damping (DFT-D3BJ).[S5,S6] When performing geometry optimization and frequency calculations, we utilized the 6-31+G(d,p) basis set. We carried out the single-point energy calculations using the 6-311+G(d,p) basis set. The electrostatic potential (ESP) was visualized with the assistance of the GaussView,[S7] version 6.0 program. The reduced density gradient (RDG) analysis for non-covalent interactions was assessed using the Multiwfn and VMD software. The electron localization function (ELF) was used to analyze the degree of electron localization and the type of bonding. It involved structure optimization and self-consistent calculations to visualize electron localization.

Cell lines

The HCT116, AML12 and NCM460 cell lines were provided by the Second Affiliated Hospital of Zhejiang University. HCT116 cells were cultured in McCoy's 5A medium (FuHeng Biology, Shanghai, China) with 10% fetal bovine serum (FBS). AML12 and NCM460 cells were maintained in DMEM medium (Gibco, Shanghai, China) supplemented with 10% FBS (Grand Island, USA). Both cell lines were incubated in a humidified incubator at 37 °C with 5% CO_2.

DNA gel electrophoresis assay

To evaluate the protective effect of **KMOF-1** on the VEGF aptamers against nuclease degradation, a DNA gel electrophoresis assay was performed. The VEGF aptamers was incubated in the presence or absence of **KMOF-1**, with and without FBS as a source of nucleases. Samples were incubated at 37 °C for 1 h. Following incubation, the samples were mixed with a loading dye and loaded onto an agarose gel. Electrophoresis was conducted at a constant voltage in Tris-acetate-EDTA (TAE) buffer. The gel was then stained with ethidium bromide and visualized using a BIO-RAD imager (Bio-Rad, USA).

Hemolytic tests

Briefly, 1 mL of mouse blood was divided into five samples, which were centrifuged at 3500 rpm for 5 min and then washed three times with normal saline. Subsequently, the samples to be tested were mixed with each blood sample. After incubation at 37 °C for 30 min, all samples were centrifuged at 3500 rpm for 5 min. The absorbance of the liquid supernatant at 540 nm was measured using a microplate reader (BioTek, Burlington, USA). PBS served as the negative control, and deionized water served as the positive control.

MTT proliferation assay

The proliferation of normal cells was examined by MTT assay. Cells (8×10^3) were seeded in 96-well plates until they became completely adherent. Then cells were processed with different concentrations of **KMOF-1@VEGF** for 48 h. After 48 h, 20 μL MTT (5 mg/mL) was added to each well, and the cells were incubated for an additional 4 h at 37 °C. The MTT solution was then removed and 100 μL of dimethyl sulfoxide (DMSO) was added to dissolve the crystals. The absorbance was determined at 490 nm wavelength with a microplate reader (BioTek, Burlington, USA).

Statistical analysis

Data were expressed in mean ± SD, or as a percentage where appropriate. Paired *t*-tests or one-way ANOVA were used where appropriate. The *p*-values are indicated in the figures.

2. *Local ESP, RDG, and ELF analyses of* ***KMOF-1***

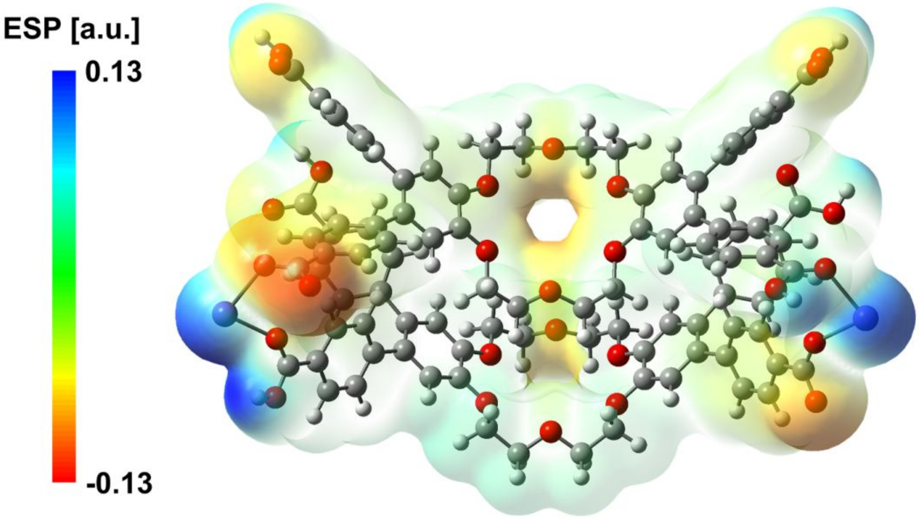

Figure S1. ESP Analysis of top-down stacking layers in **KMOF-1**.

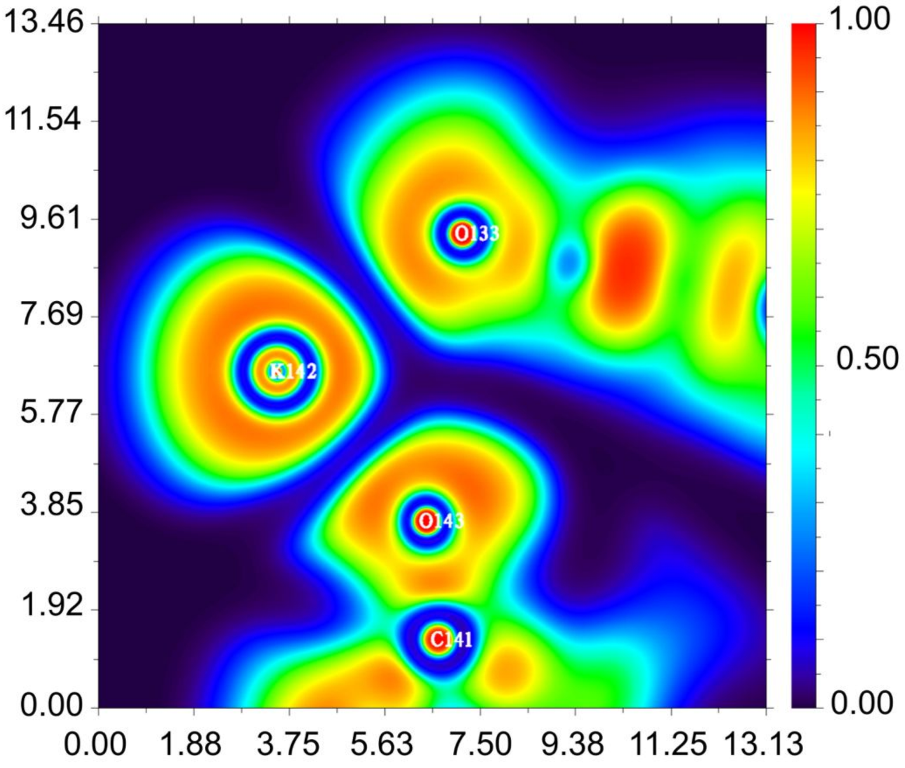

Figure S2. 2D-ELF For local top-down stacking layers in **KMOF-1**.

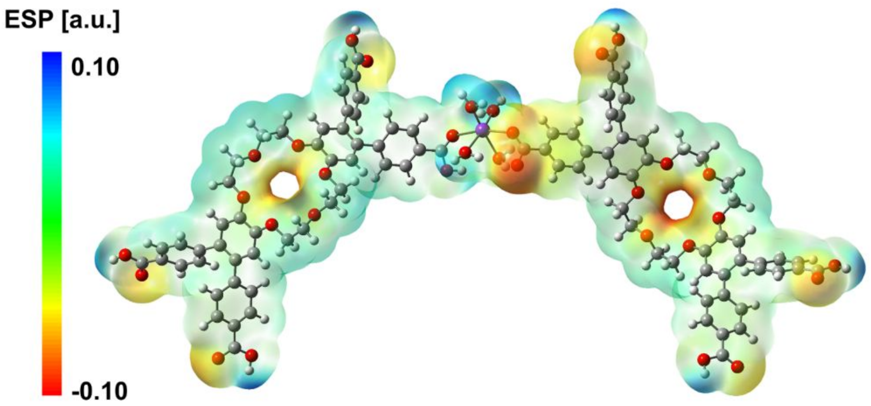

Figure S3. ESP Analysis of local left-right stacking layers in **KMOF-1**.

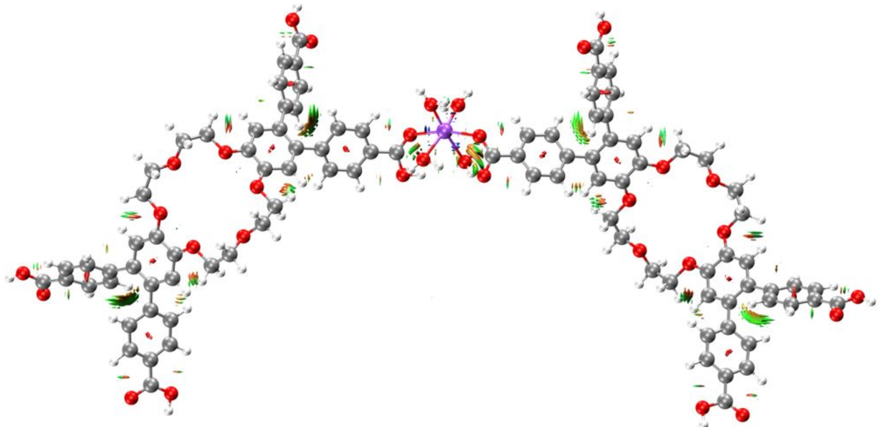

Figure S4. RDG Isosurface plot of local left-right stacking layers in **KMOF-1**.

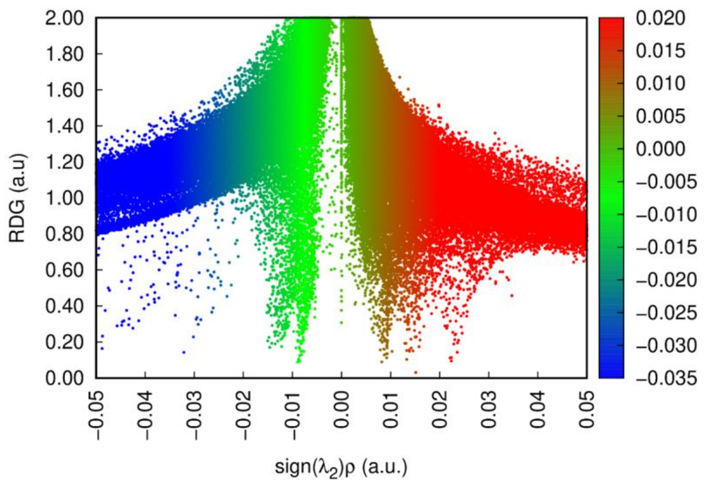

Figure S5. RDG Scatter plot of local left-right stacking layers in **KMOF-1**.

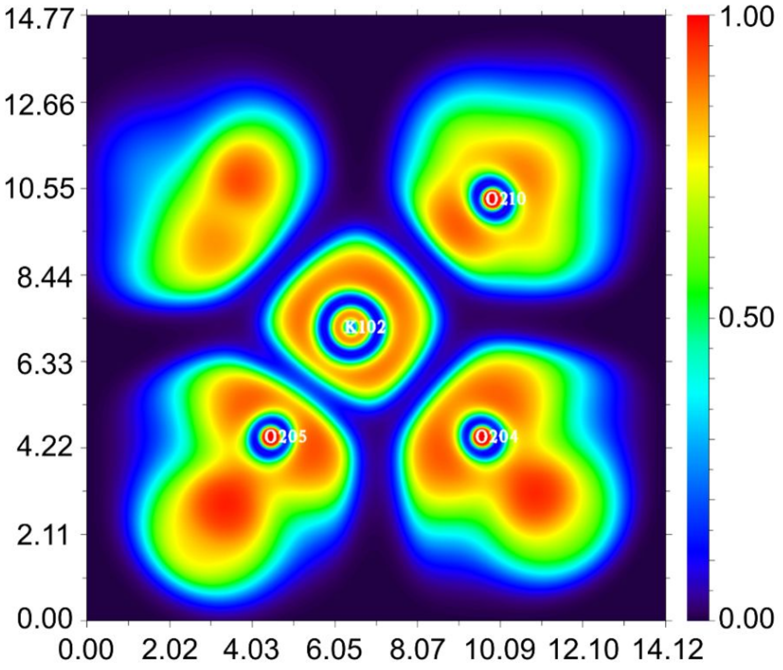

Figure S6. 2D-ELF Of local left-right stacking layers in **KMOF-1**.

3. *Stability of **KMOF-1** in different solvents*

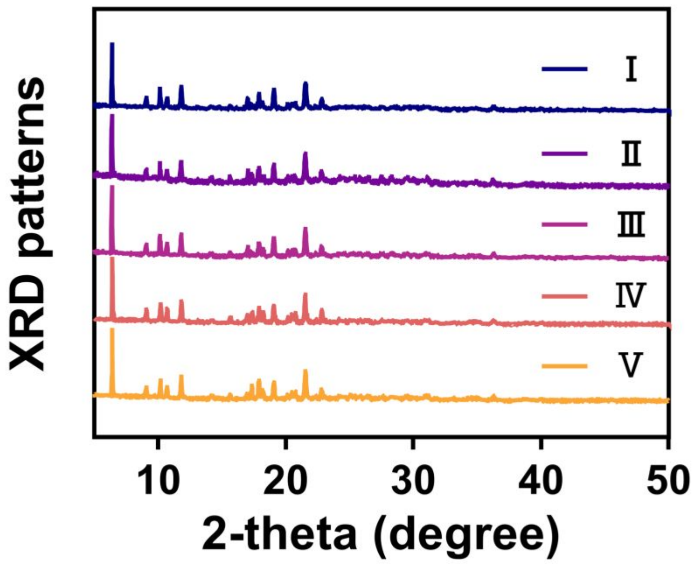

Figure S7. XRD Patterns of **KMOF-1** before and after exposure to different solvents for 24 h. I: **KMOF-1**; II: **KMOF-1** exposure to water; III: **KMOF-1** exposure to culture medium. IV. **KMOF-1** exposure to PBS buffer solution (pH = 7.4); V: **KMOF-1** exposure to PBS buffer solution (pH = 6.5).

4. *Standard curves of different drugs*

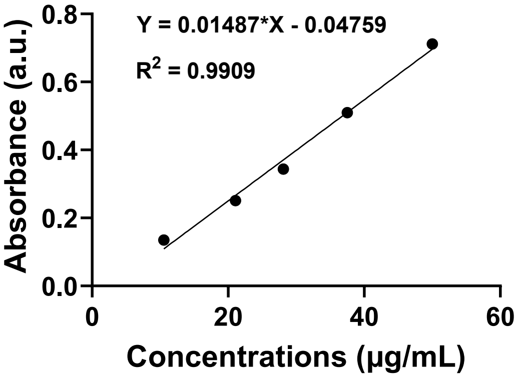

Figure S8. Standard curve of doxorubicin solutions.

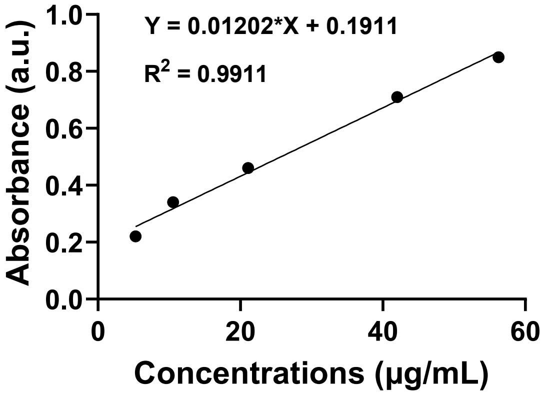

Figure S9. Standard curve of dasatinib solutions.

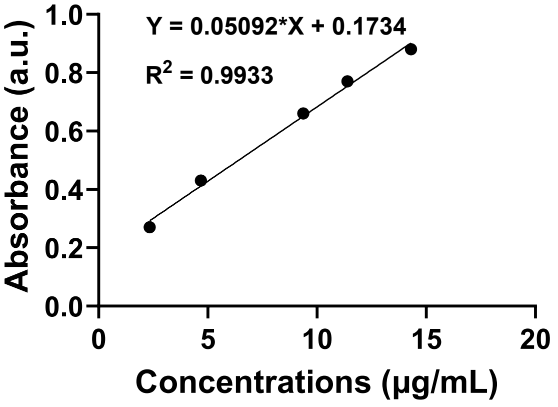

Figure S10. Standard curve of lenvatinib solutions.

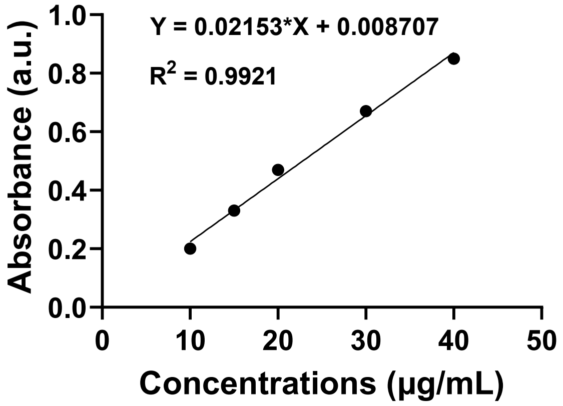

Figure S11. Standard curve of quercetin solutions.

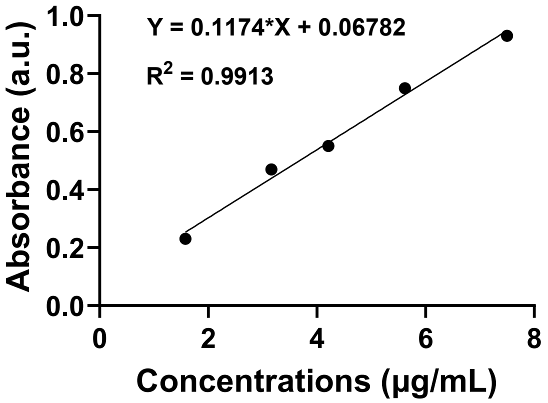

Figure S12. Standard curve of ellagic acid solutions.

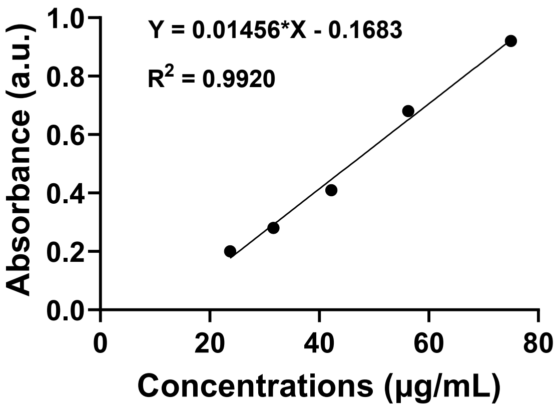

Figure S13. Standard curve of simvastatin solutions.

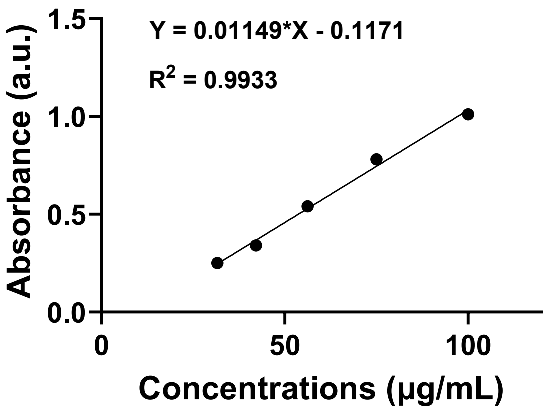

Figure S14. Standard curve of irinotecan solutions.

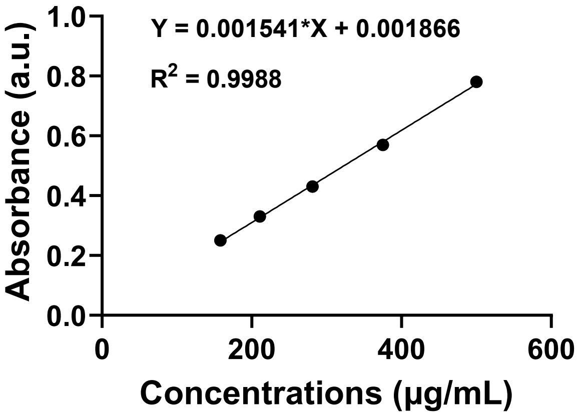

Figure S15. Standard curve of thymopentin solutions.

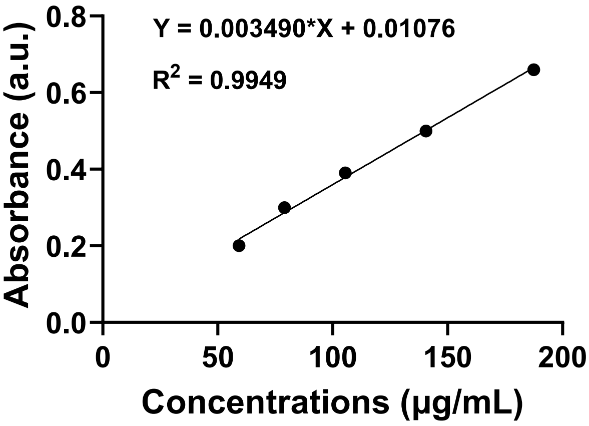

Figure S16. Standard curve of vancomycin solutions.

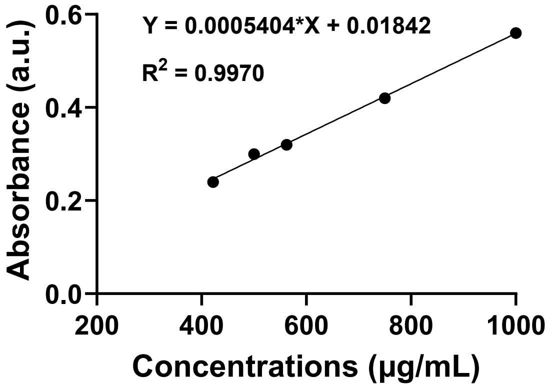

Figure S17. Standard curve of insulin solutions.

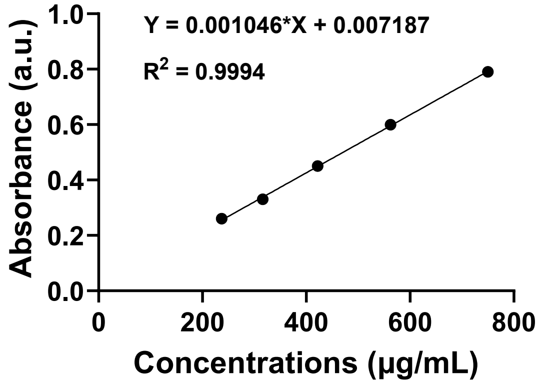

Figure S18. Standard curve of hemoglobin solutions.

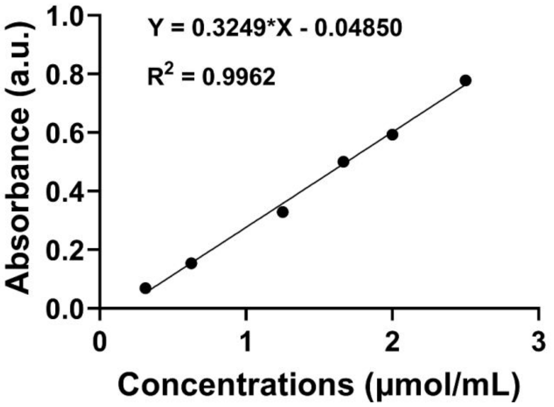

Figure S19. Standard curve of VEGF aptamer solutions.

5. *Screening of model drugs for loading*

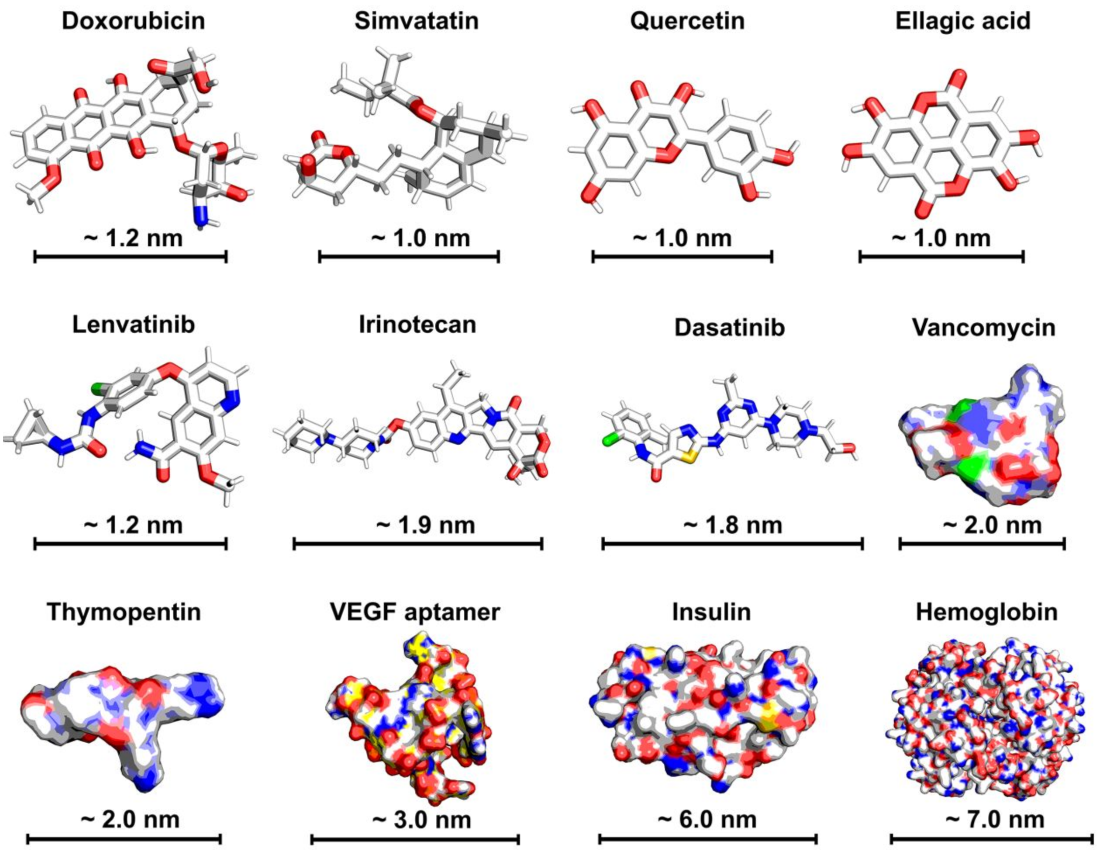

Figure S20. The maximum atomic distance between different small molecules.

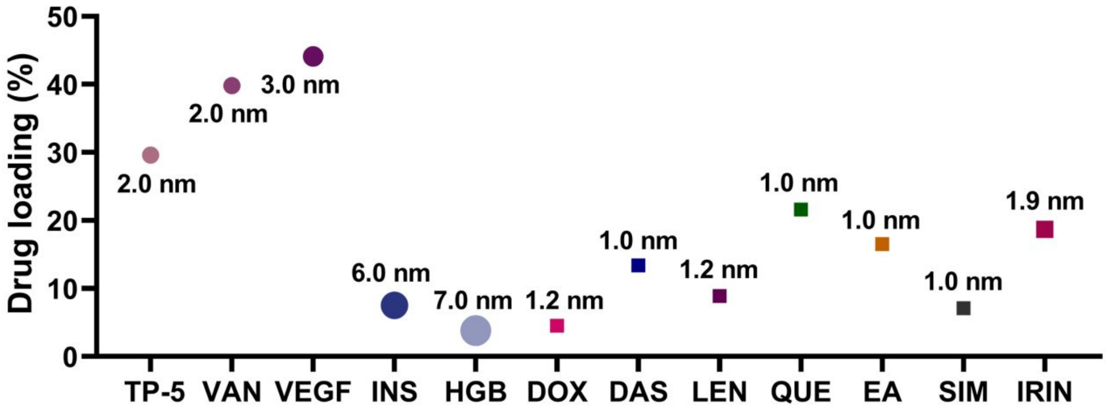

Figure S21. The drug loading capacity of **KMOF-1** for different drugs was determined at a feed ratio of 1:1 using the impregnation method.

6. *Morphological characterization of* ***KMOF-1@VEGF***

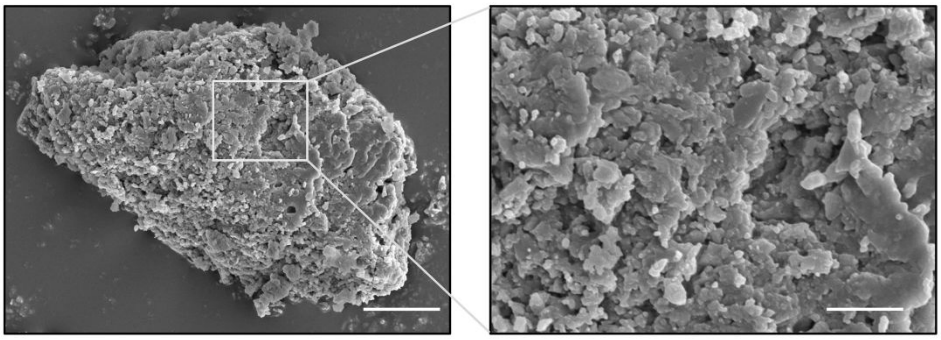

Figure S22. SEM Image of **KMOF-1@VEGF**. Bars indicate 5 μm (left panel) and 1.5 μm (right panel), respectively.

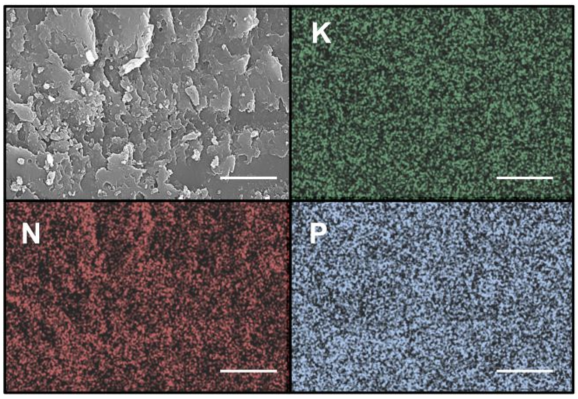

Figure S23. Elemental mapping analysis of **KMOF-1@VEGF**, confirming the presence of K, N, and P elements. Bars indicate 1.5 μm.

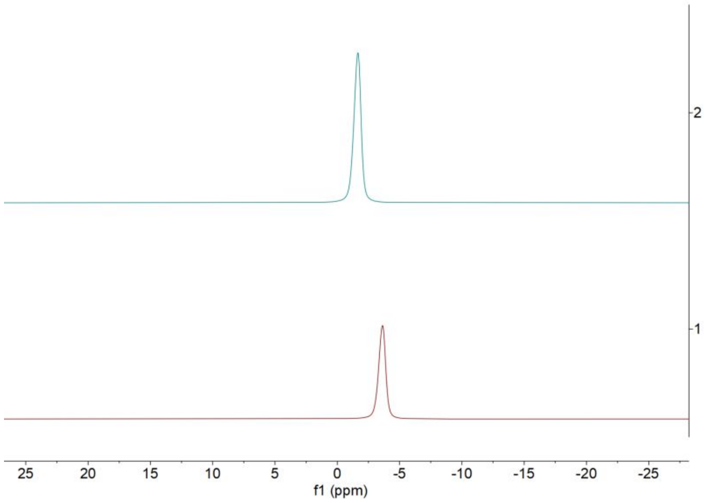

Figure S24. Solid-state ^{31}P NMR spectra (8 KHz) of VEGF (upper panel) and **KMOF-1@VEGF** (lower panel).

7. *Optimization of the drug loading capacity of* ***KMOF–1***

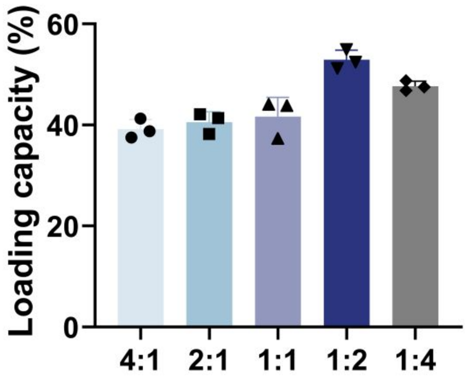

Figure S25. Loading capacity of VEGF aptamers on **KMOF-1** at different mass ratios (**KMOF-1**:VEGF).

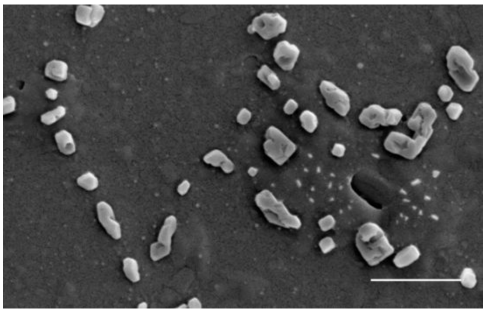

Figure S26. SEM image of **KMOF-1** after being exposed to diluted hydrochloric acid (pH < 2) for 6 h. Bars indicate 2 μm.

8. *Cell cytotoxicity of* ***KMOF-1@VEGF***

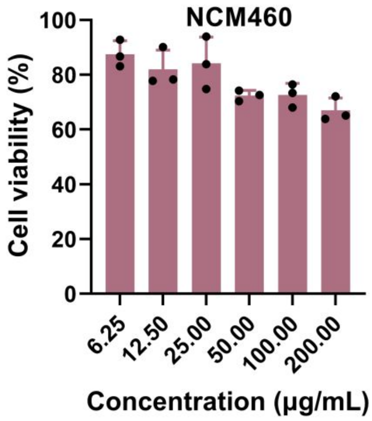

Figure S27. Cytotoxicity of **KMOF-1@VEGF** on the normal cell line NCM460.

9. *Details of crystal data*

Table S1. Crystal data and structure refinement for **KMOF-1**

Compounds	**KMOF-1**
CCDC	2402612
Empirical formula	$C_{48}H_{43}O_{16}K$
Temperature (K)	193.00
Formula weight	914.9
Crystal system	Tetragonal
Space group	$I\bar{4}$
a (Å)	44.968 (6)
b (Å)	44.968 (6)
c (Å)	9.8693 (19)
α (°)	90
β (°)	90
γ (°)	90
Volume (Å^3)	19957 (7)
Z	8
ρ_{calc} (g cm^{-3})	0.609
Absorption coefficient (mm^{-1})	0.489
F (000)	3824.0
F' (000)	3836.87
Radiation (Å)	1.34139
R (reflections)	0.0442 (8675)
wR_2 (reflections)	0.1237590)

10. Data on drug encapsulation in KMOFs

Table S2. Comparison of different KMOFs

MOFs	Organic linker	Surface area ($m^2 g^{-1}$)	Inner pore size (Å)	Drug	Drug loading (%, w/w)	Ref
CD-MOF-1	γ-CD	786	19	Ibuprofen	13	S8
CD-MOF-2	γ-CD	157	1.7	Limonene	17	S9
CD-MOF-4	γ-CD	274	1.7	Limonene	/	S9
CD-MOF-8	γ-CD	469	1.7	Limonene	/	S9
K-CD-MOFs	β-CD	/	1.7	Emodin	19	S10
α-CD-MOFs	α-CD	/	1.2	5-Fu	25	S11
γ-CD-MOFs	γ-CD	624	1.7	Myricetin	46	S12
**KMOF-1**	Crown derivative	1034	3.3	VEGF	52	This study

11. References

[S1] G. M. Sheldrick, *Acta Cryst. C* **2015**, *71*, 3–8.

[S2] O. V. Dolomanov, L. J. Bourhis, R. J. Gildea, J. A. K. Howard, H. Puschmann, *J. Appl. Cryst.* **2009**, *42*, 339–341.

[S3] A. L. Spek, *Acta Cryst. C* **2015**, *71*, 9–18.

[S4] W. Hu, B. Ye, G. Yu, H. Yang, H. Wu, Y. Ding, F. Huang, W. Wang, Z. Mao, *Adv. Sci.* **2024**, *11*, e2305382.

[S5] C. Adamo, V. Barone, *J. Chem. Phys.* **1999**, *110*, 6158–6170.

[S6] S. Grimme, S. Ehrlich, L. Goerigk, *J. Comput. Chem.* **2011**, *32*, 1456–1465.

[S7] T. Lu, Q. X. Chen, *J. Comput. Chem.* **2022**, *43*, 539–555.

[S8] H. Li, N. Lv, X. Li, B. Liu, J. Feng, X. Ren, T. Guo, D. Chen, J. Fraser Stoddart, R. Gref, J. Zhang, *Nanoscale* **2017**, *9*, 7454–7463.

[S9] Z. Qin, Q. Jiang, Y. Zou, M. Chen, J. Li, Y. Li, H. Zhang, *Small* **2024**, *20*, e2400399.

[S10] A. Yang, H. Liu, Z. Li, L. Li, W. Li, K. Liu, Polyhedron **2019**, *159*, 116–126.

[S11] J. Q. Sha, X. H. Zhong, L. H. Wu, G. D. Liu, N. Sheng, *RSC Adv.* **2016**, *6*, 82977–82983.

[S12] Y. Chen, J. Wei, Y. Chu, P. Zhu, T. Zhang, L. Mao, Y. Gao, L. Chen, F. Yuan, *Food Hydrocolloids* **2024**, *147*, 109318.
